# Beliefs About Emotion Are Tied to Beliefs About Gender: The Case of Men’s Crying in Competitive Sports

**DOI:** 10.3389/fpsyg.2019.02765

**Published:** 2019-12-13

**Authors:** Heather J. MacArthur

**Affiliations:** ^1^Department of Psychology, Hamilton College, Clinton, NY, United States; ^2^Penn State University, University Park, PA, United States

**Keywords:** emotion, adult tears and crying, gender, masculinity, femininity, competitive sports

## Abstract

Gender and emotion stereotypes suggest that men do not and should not cry, yet men’s crying seems to be particularly prominent in contexts such as competitive sports. In two studies, I investigated the possibility that men’s crying is more frequent and seen as more acceptable in these settings because such contexts are perceived to be highly masculine, and can buffer men from the negative consequences associated with violating gender stereotypes. Specifically, I tested the hypotheses that (a) observers would perceive men’s crying more positively in a masculine-stereotyped than a feminine-stereotyped setting, and following from this, (b) men would report being more likely to shed tears in a stereotypically masculine than a stereotypically feminine setting. To test these predictions, I conducted two between-subjects experiments in which participants (*N* = 250; *N* = 192), read a vignette about a man or a woman crying in either a stereotypically masculine (firefighting, weightlifting) or stereotypically feminine (nursing, figure skating) setting, and then rated the target on several emotion-related dependent variables. In line with predictions, results of Study 1 indicated that participants rated crying male firefighters as more emotionally appropriate, emotionally strong, and as higher in workplace status than crying male nurses, and that these effects were mediated by perceptions of the target’s masculinity and femininity. Study 2 replicated these effects using sports-related vignettes, and showed that male participants reported being more likely to shed tears after losing a competition in weightlifting than in figure-skating. Taken together, these findings suggest that men who are perceived to embody cultural ideals of masculinity may be given more room to cry than those who are perceived as less stereotypically masculine.

## Introduction

Previous research has demonstrated that gender plays an important role in people’s beliefs about emotion. People believe, for instance, that women express emotion more frequently and with greater intensity than men do (Fabes and Martin, 1991; Fischer, 1993). Women are also believed to experience and express a wider range of emotions than men, with almost all emotions being more readily associated with women than men (Plant et al., 2000). Crying, an expression than can be associated with multiple emotions (sadness, anger, frustration, overwhelm, feeling “moved”), has been characterized as an expression of “powerless” feelings because of its tendency to arise when other forms of action or change are not possible (Cretser et al., 1982; Vingerhoets et al., 2001). For this reason, crying has also been stereotyped as feminine, and young boys are often taught from an early age to restrain from shedding tears or showing other forms of “feminine” emotional expression (Fivush et al., 2000; Oransky and Marecek, 2009).

At the same time as tears are stereotyped as feminine and discouraged in boys and men, there are certain contexts that seem to allow men more freedom to cry without being socially penalized. One context in which it seems to be particularly acceptable for men to cry is competitive sports, where male athletes regularly shed tears after major losses, defeats, retirements, injuries, and other important moments on and off the field (Fox, 2004; Wong et al., 2011). Fox (2004) showed, for example, that sports was one of the few contexts in which men reported crying as much as women in the United Kingdom, and Wong et al.’s survey of male college football players showed that these players considered it relatively normal and acceptable to shed tears on the field. Indeed, sports appear to be a context in which men can express a range of emotions and behaviors that might otherwise be viewed as off-limits, including joy, hugging, and close physical contact with other men (Nelson, 1994; Kneidinger et al., 2001; Walton et al., 2004).

The prominence and acceptance of tears in men’s competitive sports may seem surprising, even counter-intuitive, given the historical association of sports with dominant forms of masculinity (e.g., Messner, 1992). Indeed, numerous studies have shown that male athletes are regarded as heroic representations of ideal manhood (e.g., Goodman et al., 2002; Connell and Messerschmidt, 2005), and that attributes seen as important in sports, such as competitiveness, aggression, physical strength, and stoicism, are also those perceived to be crucial in enacting Western conceptions of masculinity (Messner, 1992; Mahalik et al., 2003; Wong et al., 2011). Given this association of men’s competitive sports with masculinity, toughness, and aggression, it might be expected that stereotypical expectations for the expression of emotion would be even more strictly enforced in this context than in other areas of everyday life.

Drawing from theory in feminist masculinity studies, however, I suggest here (as elsewhere, see MacArthur and Shields, 2015; MacArthur and Shields, in press), that men’s crying may be particularly prominent and public in competitive sports precisely *because* sports are perceived as highly masculine. More specifically, Connell (1995) argues that relationships among men are ordered hierarchically, with those who represent hegemonic forms of masculinity (e.g., those espousing current cultural ideals) maintaining power and dominance over men who represent subordinate forms of masculinity (e.g., those seen to be aligned with women and femininity). Although claiming membership in the privileged group affords men increased power and social status, especially over women (Schrock and Schwalbe, 2009), enacting characteristics associated with the devalued group (women) may result in particularly harsh social punishment, as evidenced by numerous studies showing that boys and men who violate gender stereotypes are punished more harshly than girls and women who do (e.g., Levy et al., 1995; Sirin et al., 2004). Consistent with these ideas, the precarious manhood hypothesis (Vandello and Bosson, 2013) posits that because manhood is a valued social status, it is also viewed as one that must be earned through consistent manhood acts, whereas womanhood is ascribed and viewed as a biological inevitability (Vandello and Bosson, 2013).

Given these perspectives on masculinity and the importance for men of earning one’s place within the hierarchy, I predict that crying will be viewed most positively when enacted by men who, by virtue of their success in masculine domains that require characteristics such as strength, are seen as having earned their manhood. In other words, to the extent that a man is perceived as strong and stereotypically masculine, he may be allowed to express emotion that would otherwise be deemed as weak or feminine. Further, given the often heroic status of men who are seen to embody hegemonic ideals (Goodman et al., 2002), tears shed by such men may not only be tolerated, but perhaps even interpreted as a sign of strength (Lutz, 1999). It also follows that, if observers view men’s tears more favorably when shed in a highly masculine context, perhaps men might also feel more comfortable shedding tears themselves in such a context.

In the two studies presented below, I tested the hypothesis that men’s crying would be deemed most acceptable in contexts that are perceived as masculine (Study 1), and that men would report greater likelihood of crying in a stereotypically masculine than in a stereotypically feminine setting (Study 2). In both studies, I also wanted to examine whether men’s crying would be evaluated differently than women’s crying in stereotypically masculine and stereotypically feminine contexts. Previous research comparing perceptions of women’s and men’s crying has shown mixed results, with some studies finding that men’s tears are judged more favorably than women’s (Labott et al., 1991; Warner and Shields, 2007), others showing the opposite pattern (e.g., Fischer and Manstead, 1998; Fischer, 2006, as cited in Cretser et al., 1982; Fischer et al., 2004), and still others showing no differences (Hendriks et al., 2008; Brooks, 2011; Zawadzki et al., 2013). Given these inconsistent findings, I wanted to examine the perceived masculinity and femininity of the context as a potential moderator of how men’s and women’s tears are viewed.

## Study 1

In Study 1, I examined observers’ perceptions of crying targets in a stereotypically masculine (firefighting) versus stereotypically feminine (nursing) occupational context. I began with these two occupations because they are both clear-cut examples of stereotypically feminine and masculine jobs: nursing employs primarily women and is perceived to be feminine (e.g., Liben and Bigler, 2002; O’Connor, 2015), while firefighting employs primarily men and is thought to have a highly masculine culture (e.g., Hall et al., 2007; Khan et al., 2017). At the same time, firefighting and nursing are comparable because they are both helping occupations that involve medical intervention, and sociological research has shown that they are viewed similarly in terms of their occupational prestige (Smith and Son, 2014). In Study 1, then, participants read a vignette about a male or female protagonist crying in the context of either firefighting or nursing, and rated the protagonist on a number of emotion-related variables.

### Hypotheses

#### Hypothesis 1: Effect of Occupation

In Hypothesis 1, I predicted that participants would perceive crying firefighters more favorably (e.g., as more emotionally appropriate and emotionally strong) than crying nurses overall. I expected, however, that this effect would be driven by ratings of male targets, whose crying would be seen more favorably when enacted by men perceived to have earned a high degree of masculine status (firefighters) than when enacted by men perceived to have earned less masculine status (nurses). I describe this occupation by target gender interaction below.

#### Hypothesis 2: Occupation by Target Gender

The research cited above on the precarious manhood hypothesis suggests that men may be both uniquely rewarded for enacting masculine expectations and uniquely punished for failing to enact these expectations, while women are more automatically afforded status as women. Therefore, I predicted that participants’ perceptions of crying firefighters and nurses would differ based on target gender. Specifically in Hypothesis 2a, I predicted that because masculinity is culturally valued (Connell, 1995), and because male firefighters are seen as heroes and exemplars of idealized masculinity in North American society (e.g., Tracy and Scott, 2006; Hall et al., 2007), crying male firefighters would be rated more favorably than both crying male nurses and crying female firefighters across the dependent variables. Similarly, given that femininity is culturally devalued, particularly in men (Connell, 1995), I predicted in Hypothesis 2b that a crying male nurse would be rated more negatively than both crying male firefighters and crying female nurses across the dependent variables.

As an exploratory analysis, I also wanted to examine whether participants would perceive female firefighters and female nurses differently across the DVs. Given that women are sometimes punished for violating gender roles and other times rewarded for enacting masculine characteristics (e.g., Glick et al., 1988; Dodge et al., 1995; Heilman et al., 1995; Rudman and Phelan, 2008), I did not make specific predictions about whether crying female firefighters or nurses would be rated most favorably on the emotions-related dependent variables.

#### Hypothesis 3: Mediation

In Hypothesis 3, I predicted that perceptions of the crying individual’s masculinity and femininity would mediate the relationship observed between occupation (firefighting/nursing) and the dependent variables (e.g., emotional appropriateness) when participants rated male vignette targets.

Specifically, in Hypothesis 3a, I predicted that male firefighters would be perceived as more masculine than male nurses, and that these ratings of the target’s masculinity would in turn predict participants’ ratings on the dependent variables, such that male targets rated as more masculine would be perceived as more emotionally appropriate, emotionally strong, etc.

Similarly, in Hypothesis 3b, I predicated that femininity would mediate the relationship in the opposite direction: I expected that male firefighters would be perceived as less feminine than male nurses, and that the male target’s perceived femininity would negatively predict ratings of the target on the dependent variables.

As an exploratory analysis, I also wanted to examine whether this relationship would be present when participants rated female targets. If present, I predicted in Hypothesis 3c that this indirect relationship would be stronger when participants rated crying male targets than when they rated crying female targets.

### Method

#### Participants and Design

To determine how emotion is perceived when expressed by women and men in different occupational contexts, I conducted a 2 [occupation: masculine (firefighting)/feminine (nursing)] × 2 (target gender: female/male) between-subjects experiment. After receiving IRB approval for the project, participants (*N* = 255) were recruited from the psychology participant pool at a large public university in the northeastern United States, and received course credit for their participation. Sample size was based on an *a priori* power analysis using G^∗^Power 3 (Faul et al., 2007), with power set at 0.8 and alpha set at 0.05. Because no published studies had examined perceptions of men’s and women’s crying in different contexts at the time that the research was conducted, I conservatively estimated based on my own past research on gender that effect sizes would fall in the medium-small range, and set effect size at η_*p*_^2^ = 0.03 (small). The power analysis revealed that a sample size of 256 would be adequate to test the anticipated effects.

Given that the meaning of emotions and emotion-related words may differ across cultures and languages (e.g., Becht and Vingerhoets, 2002), recruitment was limited to United States citizens who spoke English as a first language. Five participants were excluded for failing two or more of three attention checks that asked them to select a particular response (e.g., “for this question, please select 7”). The final sample consisted of 250 participants (52% female, 48% male), who identified primarily as White (83.6%), followed by Asian (6.4%), mixed race (4.0%) Black or African American (2.4%), and Latino/a (1.2%), other (1.2%), and Middle Eastern or Arab (0.8%).

#### Procedure

Participants recruited to take part in the study were directed to an online Qualtrics survey. After providing informed consent and being told that they were participating in a study about students’ perceptions of people in difficult situations, they read one of four randomly assigned vignettes describing a male or female firefighter or nurse, who cried over an injured child encountered on the job (see Appendix A for full wording). Because race can intersect with gender in the stereotypes ascribed to women’s and men’s emotion (e.g., Donovan, 2011), and that most research on sadness and crying stereotypes has been conducted with white women and men (MacArthur and Shields, in press), I manipulated gender using the names Dan (male target) and Jessica (female target), which pilot testing (*N* = 12) by Dicicco (2013) showed to be perceived unanimously as white. After reading the assigned vignette, participants responded to the measures described below, provided some basic demographic information, and were debriefed.

#### Measures

As mentioned above, sadness and tears are viewed as “weak” emotional expressions (e.g., Cretser et al., 1982; Vingerhoets et al., 2001). Therefore, I measured dependent variables that I believed most likely to be affected by this perception: judgments of a crier’s emotional appropriateness, emotional strength, and status in the workplace. I also wanted to assess the possibility that participants would ascribe some men (and potentially women) more of the positive characteristics associated with tears (particularly when shed on behalf of a child), such as warmth and communality. I describe each of these measures in more detail below.

##### Emotional appropriateness

Perceptions of the appropriateness of the target’s emotion (e.g., “Jessica’s expression of emotion was appropriate in this situation”) were measured using four items from the perceived appropriateness subscale of Wong et al.’s (2011) Evaluations of Emotional Behaviors Questionnaire (EEBQ). Items were measured on a 7-point Likert scale ranging from “Strongly disagree” to “Strongly agree.” All items were summed and averaged to create a single emotional appropriateness variable (α = 0.81), in which higher scores represented greater perceived emotional appropriateness.

##### Emotional strength

This *ad hoc* measure consisted of three items that tap into how mentally and emotionally strong a target is perceived to be. These were, “How strong is Jessica, mentally and emotionally?,” “How tough is Jessica, mentally and emotionally,” and “How weak is Jessica, mentally and emotionally” (reverse scored). All items were measured on a 7-point Likert scale ranging from “Not at all” to “Extremely.” Items were summed and averaged to create a single emotional strength variable (α = 0.87), in which higher scores represented greater perceived emotional strength.

##### Workplace status

Workplace status was a three-item *ad hoc* measure that assessed how good the target was perceived to be at their job, and how much status they were imagined to hold in the eyes of their coworkers. The three items included: “How good do you think Jessica is at her job?,” “In general, how respected do you think Jessica is within her job?,” and “How much authority and status do you think Jessica has in the eyes of her coworkers?.” All items were measured on a 7-point Likert scale ranging from “Not at all/none” to “Extremely/a great deal.” Items were summed and averaged to create a single workplace status variable (α = 0.87), in which higher scores represented greater perceived workplace status.

##### Warmth

Perceptions of the target’s warmth were measured using six items from Fiske et al. (2002), which asked participants to rate the target on how warm, good-natured, sincere, unfriendly (reverse coded), trustworthy, and nice they were perceived to be. All items were measured on a 7-point Likert scale ranging from “Not at all” to “Very much.” Items were summed and averaged to create a single warmth variable (α = 0.88), in which higher scores represented greater perceived warmth.

##### Communalism

The target’s perceived communalism (e.g., the extent to which they are seen as carding and concerned with the welfare of others) was measured using four items from Heilman and Okimoto (2007), which asked participants to rate the target on how supportive, sensitive, understanding, and uncaring (reverse coded) they were perceived to be. All items were measured on a 7-point Likert scale ranging from “Not at all” to “Very much.” Initial reliability analysis showed poor reliability (α = 0.54); further analysis revealed that the reverse coded item (uncaring) was largely responsible for the low alpha. After excluding this item, reliability increased to 0.74.

##### Masculinity and femininity

The perceived masculinity and femininity of the target were measured using one item for each construct: “Overall, how masculine is Dan?” and “Overall, how feminine is Dan?” For male (Dan) and female (Jessica) targets, participants were asked to rate both perceived masculinity and femininity. Both items were measured on a 7-point Likert scale ranging from “Not at all masculine/feminine” to “Extremely masculine/feminine.”

### Results

Means and standard deviations for each of the main dependent variables, separated by occupation condition, are presented in Table 1. Correlations among the dependent variables are presented in Table 2, and full data for Study 1 are available as a supplement (see Supplementary Table S1). To evaluate my hypotheses, I conducted a two-way ANOVA on each dependent variable, using target occupation and target gender as between-subjects factors in the analysis.

**TABLE 1:** Study 1 means and standard deviations separated by occupation.

	**Mean (*SD*)**		
	**Overall**	**Firefighting**	**Nursing**
	**(*N* = 250)**	**(*n* = 124)**	**(*n* = 126)**
Emotional Appropriateness	5.64	5.80 (1.13)	5.48 (1.15)
Emotional Strength	4.39	4.60 (1.24)	4.22 (1.09)
Workplace Status	5.10	5.34 (1.20)	4.86 (1.07)
Warmth	6.08	6.15 (0.72)	6.00 (0.78)
Communalism	5.89	5.91 (0.83)	5.74 (0.85)

**TABLE 2:** Correlations among dependent variables in Study 1.

	**Correlations**				
	**1**	**2**	**3**	**4**	**5**
(1) Emotional Appropriateness	–				
(2) Emotional Strength	0.34^∗∗^	–			
(3) Workplace Status	0.44^∗∗^	0.54^∗∗^	–		
(4) Warmth	0.20^∗∗^	0.05	0.28^∗∗^	–	
(5) Communalism	0.12	–0.01	0.14^∗^	0.69^∗∗^	

*^∗^*p* < 0.05 and ^∗∗^*p* < 0.01.*

#### Effect of Occupation

In Hypothesis 1, I predicted that crying firefighters would be rated more positively (e.g., as more emotionally appropriate, emotionally strong, warm, communal, and higher in workplace status) than crying nurses across the dependent variables. ANOVA results largely supported this hypothesis: firefighters were rated significantly higher than nurses on emotional appropriateness, *F*(1,246) = 5.39, *p* = 0.021, η_*p*_^2^ = 0.021, emotional strength, *F*(1,243) = 6.01, *p* = 0.015, η_*p*_^2^ = 0.024, and workplace competence, *F*(1,246) = 10.92, *p* = 0.001, η_*p*_^2^ = 0.042. No significant main effect of occupation was found for warmth, *F*(1,243) = 2.29, *p* = 0.132, η_*p*_^2^ = 0.009 or communalism, *F*(1,246) = 0.01, *p* = 0.943, η_*p*_^2^ < 0.000.

#### Occupation × Target Gender

In Hypothesis 2, I predicted an occupation by target gender interaction. Specifically, I expected in Hypothesis 2a that participants would rate a crying male firefighter more positively than both a crying male nurse and a crying female firefighter, and in Hypothesis 2b that participants would rate a crying male nurse more negatively than both a crying male firefighter and a crying female nurse. ANOVA results, however, did not support this hypothesis, as no interaction emerged between occupation and target gender for any of the DVs, all *p*’s > 0.05.

Although the interaction between occupation and target gender was not significant, as an exploratory analysis, I conducted *post hoc* tests to determine whether differences in participants’ judgments of firefighters and nurses (e.g., the main effect of occupation) held true for both male and female targets. Results indicated that while male firefighters were judged as more emotionally appropriate, *F*(1,246) = 5.27, *p* = 0.023, η_*p*_^2^ = 0.021, more emotionally strong, *F*(1,243) = 6.19, *p* = 0.014, η_*p*_^2^ = 0.025, and higher in workplace status, *F*(1,246) = 13.99, *p* < 0.001, η_*p*_^2^ = 0.054, than male nurses, female firefighters did not significantly differ from female nurses on ratings of emotional appropriateness, *F*(1,246) = 1.02, *p* = 0.314; η_*p*_^2^ = 0.004, emotional strength, *F*(1,243) = 1.01, *p* = 0.315, η_*p*_^2^ = 0.004, or workplace status, *F*(1,246) = 0.95, *p* = 0.330, η_*p*_^2^ = 0.004.

#### Mediation

**FIGURE 1:**
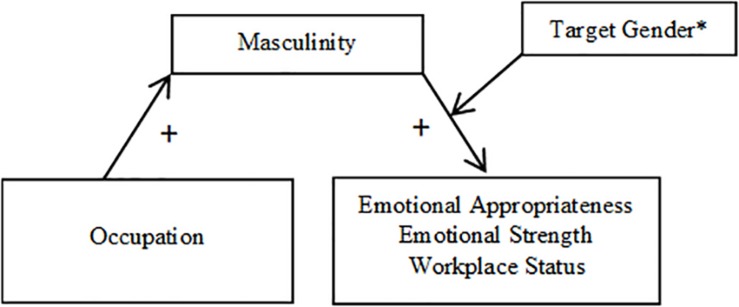
Mediation model using masculinity as a mediator (Study 1). ^∗^Indicates that the pathway between masculinity and the DVs was either significant only when participants rated male targets (emotional appropriateness, workplace status), or the relationship was stronger when participants rated male targets than when they rated female targets (emotional strength).

**FIGURE 2:**
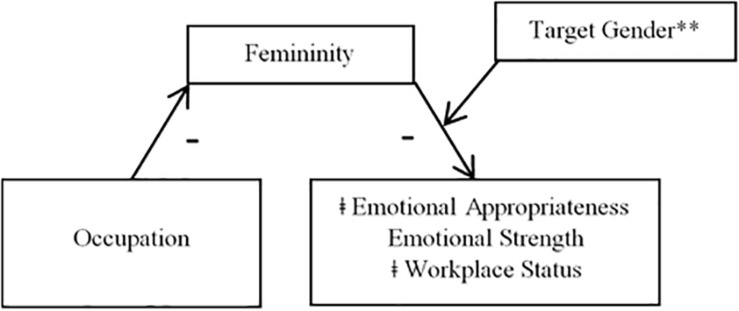
Mediation model using femininity as a mediator (Study 1). ^∗∗^Indicates that the pathway between femininity and the DVs was significant only when participants rated male targets and not female targets. ‡Indicates that the effect of occupation was still significant after the interaction between femininity and target gender was included in the model (partial mediation).

In Hypothesis 3, I predicted that perceptions of the target’s masculinity (Hypothesis 3a) and femininity (Hypothesis 3b) would mediate the relationship between occupation and the dependent variables, particularly when participants rated male targets. To test the hypothesized models, I used the PROCESS macro for SPSS (Hayes, 2012). I entered occupation (nursing = 0, firefighting = 1) as the predictor variable, perceived masculinity and femininity (in turn) as the mediators, and emotional appropriateness, emotional strength, and workplace status (in turn) as outcome variables. Given that there was no significant main effect of occupation on warmth or communalism, these variables were not included in the analyses. To determine whether masculinity and femininity mediated the relationship between occupation and the DVs differentially for male and female vignette targets (Hypothesis 3c), I selected Model 14 (moderated mediation; see Figures 1, 2) and used 5000 bootstrap samples.

##### Masculinity

For analyses of Hypothesis 3a with masculinity as a mediator, indices of moderated mediation using Model 14 were significant for emotional appropriateness (CI = 0.04,0.45), emotional strength (CI = 0.08,0.54), and workplace status (CI = 0.03,0.47). Occupation significantly predicted the perceived masculinity of the target, *F*(1,248) = 11.25, *p* = 0.001, such that firefighters (*M* = 4.12, *SD* = 1.71) were perceived to be significantly more masculine than nurses (*M* = 3.42, *SD* = 1.59). An interaction between masculinity and target gender significantly predicted all three outcome variables: emotional appropriateness, *t*(1,245) = 2.79, *p* = 0.006, emotional strength, *t*(1,242) = 3.71, *p* < 0.001, and workplace status, *t*(1,245) = 2.94, *p* = 0.004. For emotional appropriateness and workplace status, the nature of this interaction was such that the indirect effect of occupation through perceived masculinity was significant only for participants rating male targets, and not those rating female targets. For male targets, those perceived to be more masculine were also rated higher on emotional appropriateness and workplace competence, whereas masculinity did not significantly predict emotional appropriateness or workplace status for female targets. Specifically, for ratings of male targets, the 95% confidence interval for emotional appropriateness (CI = 0.07,0.41) and workplace status (CI = 0.10,0.56) did not include zero, whereas for ratings female targets, the 95% confidence interval for emotional appropriateness (CI = −0.12,0.13) and workplace status (CI = −0.02,0.24) did contain zero.

For emotional strength, the interaction revealed that masculinity was a significant mediator of the relationship between occupation and emotional strength when participants rated both male (CI = 0.15,0.67) and female targets (CI = 0.01,0.27), such that both male and female targets were rated as more emotionally strong when they were seen as more masculine. However, this indirect effect was stronger when participants rated male targets than when they rated female targets.

The direct effect of occupation was no longer significant once the interaction between masculinity and target gender was accounted for in all three models: emotional appropriateness, *t*(1,245) = 1.46, *p* = 0.144, workplace status, *t*(1,245) = 1.85, *p* = 0.066, and emotional strength, *t*(1,242) = 0.65, *p* = 0.518. Thus, masculinity fully mediated the effects of occupation on all three variables.

##### Femininity

Results of analyses of Hypothesis 3b using femininity as a mediator indicated that moderated mediation effects were significant only for emotional appropriateness (CI = 0.02,0.43). The pathway from occupation to perceived femininity was significant, *F*(1,248) = 7.23, *p* = 0.008, such that nurses (*M* = 4.49, *SD* = 1.68) were rated as significantly more feminine than firefighters (*M* = 3.91, *SD* = 1.73). An interaction between femininity and target gender then significantly predicted emotional appropriateness, *t*(1,245) = −2.88, *p* = 0.005, such that the indirect effect through perceived femininity was significant only for participants rating male targets (CI = 0.01,0.26) and not those rating female targets (CI = −0.23,0.03). That is, to the extent that male targets were perceived as feminine, they were also rated as less emotionally appropriate, whereas the relationship between femininity and emotional appropriateness was non-significant when participants rated female targets. The direct effect of occupation was still significant after the interaction between femininity and target gender was accounted for in the model, *t*(1,245) = 2.30, *p* = 0.023, indicating that femininity was a partial mediator of the relationship between occupation and emotional appropriateness.

For workplace status, although the index of moderated mediation was not significant (CI = −0.05,0.31), indicating that the indirect effect of occupation through perceived femininity was not significantly different for female and male vignette targets, analyses nevertheless revealed that the moderated mediation model was significant for male targets (CI = 0.03,0.33), but not female targets (CI = −0.07,0.20). Similar to emotional appropriateness, this meant that to the extent crying male targets, but not female targets, were seen as feminine, they were also seen as having less status in their jobs. The effect of occupation remained significant after femininity was included in the model, *t*(1,245) = 2.54, *p* = 0.012, indicating a partial mediation effect.

Analyses for emotional strength revealed that perceived femininity functioned as a significant mediator for participants rating both male (CI = 0.03,0.32) and female targets (CI = 0.02,0.31); therefore I ran a simple mediation analysis using Process model 4. This analysis revealed a significant indirect effect of occupation on emotional strength through perceived femininity (CI = 0.01,0.17). Occupation significantly predicted femininity, *F*(1,245) = −2.55, *p* = 0.011, femininity significantly predicted emotional strength, *t*(1,2245) = −3.42, *p* = 0.001, such that targets rated as more feminine were also rated as less emotionally strong, and occupation was no longer a significant predictor of emotional strength once femininity was included in the model, *t*(1,245) = 1.96, *p* = 0.051. Thus, perceived femininity fully mediated the effect of occupation on emotional strength for both male and female targets.

**FIGURE 3:**
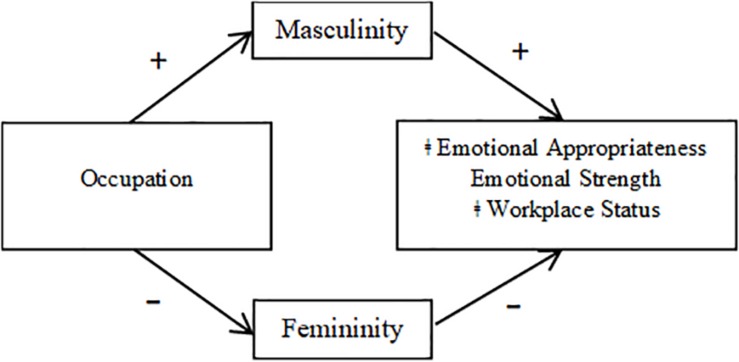
Mediation model for male targets (Study 1). ‡Indicates that femininity was a partial mediator of the relationship between occupation and emotional appropriateness and between occupation and workplace status.

**FIGURE 4:**
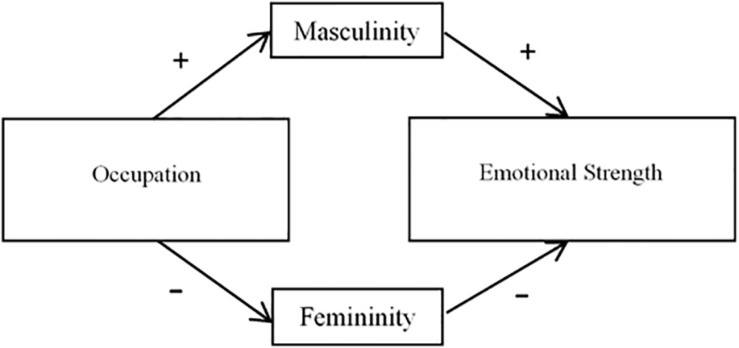
Mediation model for female targets (Study 1).

Mediation results are depicted in Figures 1, 2 (broken down by mediator), as well as Figures 3, 4 (broken down by target gender).

### Discussion

Results of Study 1 showed that while there were no differences in how participants viewed women’s crying as compared to men’s crying overall, crying firefighters were perceived as more emotionally appropriate, more emotionally strong, and as having higher workplace status than crying nurses. However, exploratory analyses indicated that these results were primarily driven by participants’ ratings of male targets: male firefighters were seen as more emotionally appropriate, emotionally strong, and as having higher workplace status than male nurses, while there were no significant differences by occupation for female targets. Mediation analyses showed that the effect of occupation on the dependent variables was fully explained by how masculine participants perceived the crier to be. Crying firefighters were perceived to be more masculine than crying nurses; masculinity in turn predicted how emotionally appropriate (male targets only), emotionally strong (male targets to a greater degree than female targets), and high in workplace status (male targets only) the vignette targets were perceived to be. Perceptions of the target’s femininity also partially explained the results in the opposite direction: crying firefighters were perceived as less feminine than nurses, and therefore were seen as more emotionally appropriate (male targets only), more emotionally strong, and as having higher status at work (male targets only).

Overall, these results support my initial hypothesis that observers would view men’s crying more favorably when that crying occurred in a stereotypically masculine rather than stereotypically feminine context: to the extent that a man is perceived as more masculine and less feminine due to his occupational role, perceivers will also rate his tears as more appropriate and acceptable. Such results align with previous theoretical and empirical work suggesting that masculinity (to a greater degree than femininity) is structured hierarchically, with dominant forms being granted more freedom and access to power than more subordinate types (Connell, 1995; Connell and Messerschmidt, 2005; de Boise, 2015).

The results are also consistent with research on the backlash effects associated with stereotype violation. Although most studies investigating stereotype violation have focused on the consequences for women, research on backlash in men has shown that men are penalized for displaying modesty in the workplace (Moss-Racusin et al., 2010), achieving success in feminine domains (Heilman and Wallen, 2010), and requesting family leave (Rudman and Mescher, 2013), to name a few examples. It is unclear, however, whether the present Study’s findings pertaining to female firefighters and nurses are consistent with this literature. That is, although female firefighters are also role violators, they do not appear to have been penalized for this in the present research. In fact, examination of means shows that female firefighters were generally viewed slightly more favorably (although not significantly so) than female nurses, and mediation analyses showed that crying women were judged as emotionally strong to the extent that they were seen as more masculine and less feminine. Perhaps this was because their tears (which are stereotype-consistent) overrode their occupational role in the eyes of participants. Regardless, findings indicate that emotional expression may be judged according to masculine standards to some degree for both men and women, and indicate that more research is needed to understand when immediate behavior (e.g., emotional expression) becomes more important than social roles in observers’ perceptions, as well as to understand the conditions in which women will be judged positively or negatively for violating gender stereotypes.

Given that no differences were found in participants’ evaluations of men’s and women’s crying in the present study, the results also align with previous research showing that men’s and women’s tears are evaluated similarly (e.g., Hendriks et al., 2008; Brooks, 2011; Zawadzki et al., 2013). Although the present study cannot explain why conflicting results have been found previously in the literature comparing perceptions of women and men’s tears, research by Fischer et al. (2013) suggests that context may play a role. In their study, crying men were seen as less competent than crying women when the crying occurred in the workplace, but there were no differences when the crying occurred in a relationship scenario. Therefore, it may be that relative to women’s crying, men’s crying is seen similarly or more positively when it occurs in a context that is generally deemed appropriate for tears (e.g., highly emotional workplace situations like tending to a seriously injured child; highly emotional personal and relationship situations), while men’s crying is downgraded relative to women’s when it occurs in contexts that are seen as less appropriate for tears (e.g., more everyday workplace events). Future research should investigate this possibility with further empirical comparisons of women’s and men’s crying in different settings.

Although the results of Study 1 provide initial evidence to suggest that men’s crying may be judged most favorably when performed in contexts and by individuals who are perceived as highly masculine, I wanted to test whether these findings would hold true when using a different vignette setting and reason for crying, and when using a non-university participant sample. Given the research cited earlier (e.g., Fox, 2004; Wong et al., 2011) suggesting that men may be particularly likely to cry in athletic contexts, I decided to use competitive sports as the setting for Study 2. To determine whether the findings of Study 1 would hold across crying scenarios, targets in Study 2 were described as crying for a self-interested reason (losing a sports competition) rather than a selfless reason (an injured child). Furthermore, to ensure that Study 1 results were not influenced by the names chosen for the vignette targets, I manipulated gender using different male and female names in Study 2.

Since results of Study 1 revealed that crying men in particular are judged for their emotional displays according to how masculine and feminine they are perceived to be, in Study 2, I wanted to extend these findings by examining whether male participants would indicate being less likely to cry in a stereotypically feminine rather than stereotypically masculine context. That is, if men in particular are likely to be judged more negatively for crying in a stereotypically feminine context, might they be inclined to keep their crying to themselves in such a setting (e.g., one that might make them appear feminine)?

## Study 2

In Study 2, I investigated how observers perceive women’s and men’s crying in a competitive sports context. I hypothesized that, as in Study 1, crying athletes would be rated more favorably in a stereotypically masculine sport (weightlifting) than in a stereotypically feminine sport (figure skating), and that these effects would be mediated by the perceived masculinity and femininity of the crying vignette target. I also hypothesized that male participants would report less likelihood of crying themselves in a figure skating versus weightlifting context, whereas the type of sport would not matter in female participants’ assessments of their likelihood of crying.

### Method

#### Participants and Design

To test my hypotheses about observers’ perceptions of crying in a sports context, I conducted a 2 [sport: masculine (weightlifting)/feminine (figure skating)] × 2 (target gender: female/male) × 2 (participant gender: female/male) between-subjects experiment. The project was IRB approved, and United States American English-speaking participants (*N* = 194) were recruited from Amazon’s Mechanical Turk. Participants were paid $0.30 for completing the survey, which took approximately 5 min. Two participants were excluded for failing an attention check that asked them to select a particular response (e.g., “for this question, please select 7”). The final sample consisted of 192 participants (52% female, 47% male). Sample size for Study 2, which was conducted when the author was a graduate student, was based on available funding for the research. A sensitivity analysis using G^∗^Power 3 (Faul et al., 2007), with power set at 0.80 and alpha set at 0.05, revealed that a sample of 192 would be sensitive enough to detect partial eta squared effect sizes equal to or greater than 0.04 (i.e., in the small effect size range). Although two of the observed effect sizes in Study 1 were smaller than this threshold, research indicates that men typically *overestimate* the extent to which others expect masculine behavior from them (Vandello et al., 2008, 2009). Therefore, a sample size of 192 was considered adequate to test the effect of my main hypothesis around men’s likelihood of refraining from feminine-stereotyped behavior in different contexts.

Female and male participants were relatively evenly split across the four vignette conditions: female figure skater (55% female, 45% male), male figure skater (45% female, 53% male), female weightlifter (58% female, 42% male), and male weightlifter (50% female, 50% male). One participant in the male figure skater condition did not indicate a gender; their data was included when analyzing participant perceptions of the vignette targets, but left out of analyses that examined the effect of participant gender on one’s own imagined reactions to the vignette scenario. The mean age of the sample was 37.61, and most participants identified as White (74%), followed by Black or African American (9.9%), Asian (7.3%), Latino/a (5.7%), Native American or Alaska Native (1.6%), and mixed race (1.6%).

#### Procedure

Participants recruited to take part in the study were directed to an online Qualtrics survey. After providing informed consent and being told that they were participating in a study about perceptions of athletes participating in various competition scenarios, they read one of four randomly assigned vignettes describing a male (Jonathan) or female (Jennifer) athlete crying over a loss in either a stereotypically masculine (weightlifting) or stereotypically feminine (figure skating) sport (see Appendix A for full vignette wording). In addition to both being individual sports, pretesting (*N* = 43) indicated that while weightlifting was perceived as significantly more masculine than figure skating, *t*(82) = 15.56, *p* < 0.001, there was no significant difference in how valued the two were perceived to be in American society, *t*(82) = 0.18, *p* = 0.858. Thus, these two sports provided ideal contexts in which to assess perceptions of athletes’ crying. After reading the vignette, participants responded to the dependent measures, provided some brief demographic information, and were debriefed.

#### Measures

Measures for Study 2 included the same scales of emotional appropriateness (α = 0.87) and emotional strength (α = 0.89) as used in Study 1. Given that the setting of Study 2 was sports rather than the workplace, and that more objective information about status was given to participants about targets in Study 2 (e.g., that they were high-level athletes competing in a national championship), workplace status was not included as a measure in Study 2. Similarly, given that no significant effects were found for warmth and communalism in Study 1, they were not included as dependent variables in Study 2. To test hypotheses about how likely participants would be to cry themselves in sports-related scenarios, a measure of emotional conformity was also included, as described below.

##### Emotional conformity

The emotional conformity measure assessed how likely participants would be to express emotion as the target did if they found themselves in a similar situation (e.g., “If I were in Jonathan’s situation, I would be likely to express emotion the way he did”). To measure this construct, I used 4 items from the emotional conformity subscale of Wong et al.’s (2011) EEBQ. Items were measured on a 7-point Likert scale ranging from “Strongly disagree” to “Strongly agree.” Items were summed and averaged to create a single emotional conformity variable (α = 0.90), in which higher scores represented greater emotional conformity, or likelihood of crying.

### Results

Means and standard deviations for each of the main dependent variables, separated by sport condition, are presented in Table 3, and correlations among the dependent variables are presented in Table 4. Full data for Study 2 are available as a supplement (see Supplementary Table S2). To evaluate my hypotheses, I conducted a three-way ANOVA on each of the dependent variables, using sport, target gender, and participant gender as between-subjects factors.

**TABLE 3:** Study 2 means and standard deviations separated by sport.

	**Mean (*SD*)**		
	**Overall**	**Weightlifting**	**Figure skating**
	**(*N* = 192)**	**(*n* = 98)**	**(*n* = 94)**
Emotional Appropriateness	5.67	5.63 (1.09)	5.71 (1.15)
Emotional Conformity	4.56	4.90 (1.33)	4.20 (1.79)
Emotional Strength	4.67	4.91 (1.21)	4.43 (1.29)

**TABLE 4:** Correlations among dependent variables in Study 2.

	**Correlations**			
	**1**	**2**	**3**	**4**
(1) Emotional Appropriateness	–			
(2) Emotional Conformity	0.57^∗∗^	–		
(3) Emotional Strength	0.49^∗∗^	0.48^∗∗^	–	

*^∗∗^*p* < 0.01.*

#### Effect of Sport

As in Study 1, I expected crying weightlifters to be rated more positively across the DVs than crying figure skaters. Replicating Study 1, ANOVA results showed that crying weightlifters were rated by participants as more emotionally strong, *F*(1,183) = 6.45, *p* = 0.012, η_*p*_^2^ = 0.034, than figure skaters, and also reported being more likely to express their own emotions like weightlifters rather than figure skaters (emotional conformity), *F*(1,183) = 10.25, *p* = 0.002, η_*p*_^2^ = 0.053. There was no significant main effect of sport on emotional appropriateness, *F*(1,183) = 0.318, *p* = 0.573, η_*p*_^2^ = 0.002.

#### Sport by Target Gender

As in Study 1, no significant interactions emerged between sport and target gender on any of the DVs, all *p*’s < 0.05. Although the interaction between target gender and occupation was not significant, I once again conducted exploratory *post hoc* analyses to determine whether differences in judgments of weightlifters and figure skaters held true for both male and female targets. Results indicated that participants reported being more likely to express their emotions like weightlifters than like figure skaters when they read about both a male, *F*(1,183) = 4.30, *p* = 0.040, and a female vignette target, *F*(1,183) = 6.03, *p* = 0.015. When the effect of sport on emotional strength was broken down by target gender, results became non-significant for both male, *F*(1,183) = 3.73, *p* = 0.055, η_*p*_^2^ = 0.02, and female, *F*(1,183) = 2.75, *p* = 0.099, η*_*p*_^2^* = 0.015, targets. Thus, results indicated that the marginal finding for male targets was likely driving the main effect of occupation observed for emotional strength.

#### Sport by Participant Gender

**FIGURE 5:**
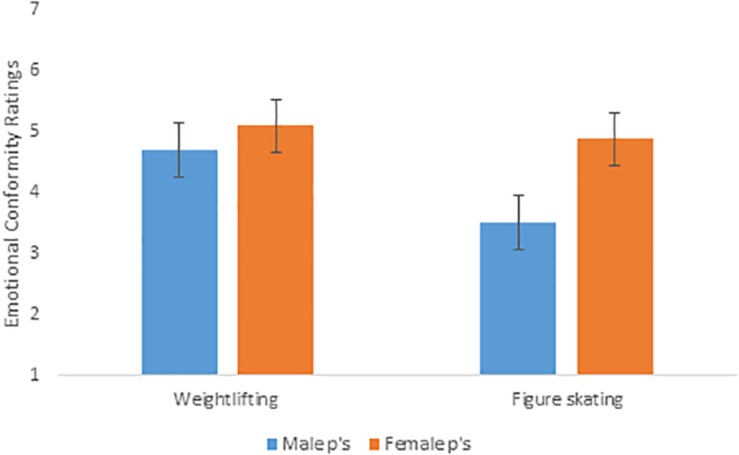
Sport by participant gender interaction on ratings of emotional conformity (Study 2).

As discussed in the introduction to Study 2, I predicted a sport by participant gender interaction on emotional conformity, such that male participants would report being more likely to express their emotion as the target did when they viewed a weightlifting rather than a figure skating vignette. In contrast, I predicted that there would be no difference in female participants’ ratings of emotional conformity based on sport condition. Consistent with these predictions, ANOVA results showed a significant interaction between sport and participant gender, *F*(1,183) = 4.82, *p* = 0.029, η_*p*_^2^ = 0.026. Pairwise comparisons showed that while there was no difference for female participants in how likely they would be to cry in weightlifting versus figure skating, *F*(1,183) = 0.528, *p* = 0.468, η_*p*_^2^ = 0.003, male participants were significantly more likely to say they would cry in weightlifting than in figure skating, *F*(1,183) = 13.957, *p* < 0.001, η_*p*_^2^ = 0.071. Similarly, while there was no difference in how likely male and female participants were to say they would cry in weightlifting, *F*(1,183) = 1.81, *p* = 0.181, η_*p*_^2^ = 0.010, female participants were significantly more likely than male participants to say they would cry in figure skating, *F*(1,183) = 19.18, *p* < 0.001, η_*p*_^2^ = 0.095. A graph of this interaction is depicted in Figure 5. No significant interaction between sport and participant gender emerged for emotional appropriateness, *F*(1,183) = 1.81, *p* = 0.181, η_*p*_^2^ = 0.010, or emotional strength, *F*(1,183) = 1.81, *p* = 0.181, η_*p*_^2^ = 0.010.

#### Mediation

As in Study 1, I predicted that perceptions of the target’s masculinity and femininity would mediate the relationship between sport and the dependent variables, especially when participants rated male targets. Using PROCESS (Hayes, 2012) model 14 with 5000 bootstrap samples, I entered sport (figure skating = 0, weightlifting = 1) as the predictor variable, perceived masculinity and femininity (in turn) as mediators, and emotional strength and emotional conformity (in turn) as the outcome variables.

##### Masculinity

Results of analyses using masculinity as a mediator revealed that indices of moderated mediation were significant for both emotional strength (CI = 0.45, 1.19) and emotional conformity (CI = 0.19, 1.08). The pathway from sport to perceived masculinity was significant, *F*(1,190) = 44.85, *p* < 0.001, such that weightlifters were perceived to be more masculine than figure skaters, and an interaction between masculinity and target gender significantly predicted emotional strength, *t*(1,187) = −5.37, *p* < 0.001, and emotional conformity, *t*(1,187) = −3.06, *p* = 0.003. The nature of both interactions was such that the indirect effect through perceived masculinity was significant only for participants rating male targets, and not those rating female targets. To the extent that male targets were rated as masculine, they were also rated higher on emotional strength and emotional conformity, whereas masculinity was not a significant predictor of emotional strength or emotional conformity for female targets. Specifically, for ratings of male targets, the 95% confidence interval for emotional strength (CI = 0.50,0.82) and emotional conformity (CI = 0.35,0.80) did not include zero, whereas for ratings of female targets, the 95% confidence interval for emotional strength (CI = −0.06,0.25) and emotional conformity (CI = −0.07,0.34) did contain zero. The direct effects of occupation were no longer significant once the interaction between target gender and the target’s perceived masculinity was accounted for in the models for emotional strength, *t*(1,190) = 0.10, *p* = 0.923, and emotional conformity, *t*(1,190) = 0.94, *p* = 0.348, indicating that the target’s perceived masculinity fully mediated the effects of occupation on both variables.

##### Femininity

Results using femininity as a mediator indicated that the index of moderated mediation was significant for both emotional strength (CI = 0.33,1.09) and emotional conformity (CI = 0.01,0.82). The pathway from sport to perceived femininity was significant, *F*(1,189) = 22.13, *p* < 0.001, such that figure skaters were perceived to be significantly more feminine than weightlifters, and an interaction between femininity and target gender significantly predicted emotional strength, *t*(1,186) = 5.06, *p* < 0.001, and emotional conformity, *t*(1,186) = 2.19, *p* = 0.03. The nature of this interaction was such that the indirect effect through perceived femininity was significant only for participants rating male targets, and not those rating female targets. Specifically, male targets who were seen as more feminine were also rated lower on emotional strength and emotional conformity, whereas femininity did not significantly predict emotional strength or emotional conformity for female targets. For male targets, the 95% confidence interval for emotional strength (CI = −0.65,−0.32) and emotional conformity (CI = −0.61,−0.18) did not include zero, whereas for female targets, the 95% confidence interval for emotional strength (CI = −0.07,0.32) and emotional conformity (CI = −0.30,0.22) did contain zero. For emotional strength, the direct effect of occupation was no longer significant once the interaction between target gender and the target’s perceived femininity was accounted for in the model, *t*(1,189) = −1.89, *p* = 0.060, indicating that the target’s perceived femininity fully mediated the effects of occupation on emotional strength. The direct effect for emotional conformity, however, was still significant after the interaction was included in the model, *t*(1,186) = −2.09, *p* = 0.038, indicating that femininity was a partial mediator of the relationship between sport and emotional conformity.

**FIGURE 6:**
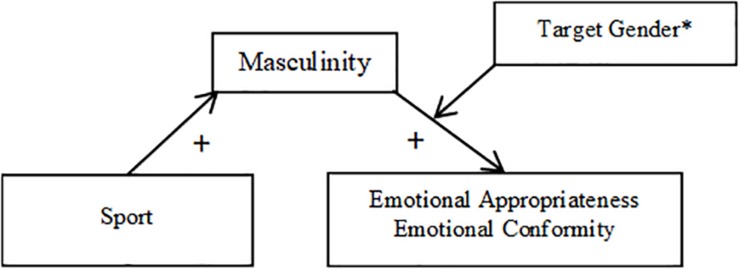
Mediation model using masculinity as a mediator (Study 2). ^∗^Indicates that the pathway between masculinity and the DVs was significant only when participants rated male targets.

**FIGURE 7:**
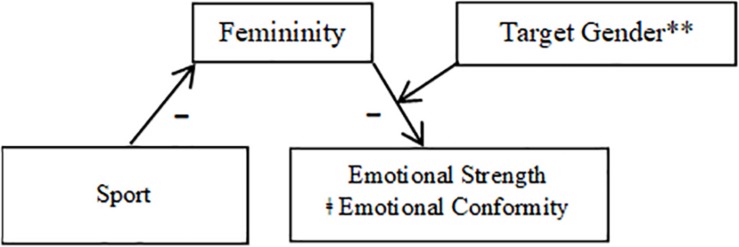
Mediation model using femininity as a mediator (Study 2). ^∗∗^Indicates that the pathway between femininity and the DVs was significant only when participants rated male targets. ‡Indicates that the effect of sport was still significant after the interaction between femininity and target gender was included in model.

Overall, mediation analyses using both masculinity and femininity as mediators were significant only for male and not for female targets; therefore, models are presented in Figures 6, 7, separated only by mediator (masculinity and femininity).

### Discussion

In Study 2, I replicated the finding from Study 1 that targets crying in stereotypically masculine settings are generally viewed more positively than those crying in stereotypically feminine settings. As in Study 1, exploratory analyses showed that these effects were largely driven by participants’ ratings of male criers. Although participants indicated greater likelihood of crying themselves (emotional conformity) when they read about a female target crying in a weightlifting rather than figure skating context, because participants were asked to imagine how they *themselves* would behave in the competition scenario, their own gender may explain this finding more than the gender of the vignette target. This interpretation is supported by the interaction that was found between sport and participant gender on emotional conformity: male participants but not female participants indicated being less likely to cry in a figure skating rather than weightlifting context, regardless of whether the vignette target they read about was a male or female athlete. Also similar to Study 1, all effects in Study 2 were mediated by perceptions of the target’s masculinity and femininity: crying male athletes in particular were viewed positively to the extent that they were also perceived as more masculine and less feminine. Thus, Study 2 findings provide further evidence that people judge men’s crying according to how masculine and feminine they perceive those men to be, and extend the results of Study 1 by showing that men themselves report being more inclined to cry in a context that is perceived as masculine. Indeed, findings for emotional conformity suggest that men may be (consciously or unconsciously) aware of observers’ tendency to rate their emotional displays according to beliefs about gender, and regulate their own displays (or reports of their imagined displays) accordingly, in this case by indicating less likelihood of crying in a stereotypically feminine context.

One difference between the results of Study 1 and Study 2 involved the fact that, in Study 1, a main effect of occupation was observed on emotional appropriateness, such that the crying of firefighters was rated as significantly more emotionally appropriate than the crying of nurses. In Study 2, however, no significant main effect of the context was found for this DV. Since vignettes in both studies depicted scenarios in which there seemed to be a valid reason for the protagonist’s tears (an injured child; loss of a national competition), it is not clear why this finding did not replicate in Study 2. One possibility is that a larger sample size was needed to capture differences in perceived appropriateness; therefore, future research using a larger sample should follow up on these conflicting findings to determine when judgments of emotional appropriateness might be affected (or not) by the context in which crying occurs.

Overall, findings of Study 2 are consistent with research cited previously showing that male athletes in stereotypically masculine sports seem to have increased freedom to display emotions that might be seen as “unmasculine” in other settings (Wong et al., 2011; MacArthur and Shields, 2015). No research to my knowledge has investigated expressions of emotion, or attitudes toward it, in sports considered less stereotypically masculine (e.g., swimming, figure skating, badminton). Thus, one interesting direction for future research would be to examine actual displays of emotion by both women and men in different sports. Given that beliefs about gender impact not only our judgments about the emotional expression of others, but also beliefs about our own expression, such a study would reveal whether such beliefs extend their influence to observable emotional behavior.

## General Discussion

Across two studies using different samples, vigette contexts, and reasons for crying, the present research showed that criers (particularly men) are seen more positively in stereotypically masculine contexts than in stereotypically feminine contexts, and that for male targets (and to a lesser extent, female targets), this effect is largely due to the fact that they are perceived as being more masculine and less feminine than those who cry in stereotypically feminine contexts. In addition, Study 2 revealed that men may be aware of the advantages of crying in a stereotypially masculine setting, in reporting that they would be more likely to cry in a stereotypically masculine versus feminine setting; no difference emerged for female targets. Overall, the results shed light on why men’s crying may be particularly prominent in settings that are perceived to be highly masculine, such as men’s competitive sports (MacArthur and Shields, 2015). Results also highlight that beliefs about emotion are fundamentally tied to beliefs about gender, both in our perceptions of others’ displays of emotion and in judgments about our own.

Taken together, the set of studies described here add to a growing body of feminist research (e.g., Warner and Shields, 2007; Wong et al., 2011; Zawadzki et al., 2013) that challenges predominant stereotypes about men’s emotional behavior. Indeed, the present research shows that popular ideas about men’s emotional inexpressiveness are misguided in both a descriptive and prescriptive sense: men do cry and can be perceived positively for it. For example, Study 2 revealed that in a weightlifting context, female and male participants were equally likely to report that they would cry after losing an important competition; only in figure skating did gender differences in crying likelihood emerge. And across both studies, mean appropriateness ratings for both women’s and men’s crying were all well above scale mid-points. Such results highlight that men’s emotional expression, and observers’ responses to it, are certainly more complex than popular stereotypes would suggest.

However, at the same time that the results challenge predominant stereotypes about men’s crying, the findings also highlight the continued importance of dominant frameworks of masculinity in people’s judgments of emotion, and in men’s reports of how likely they would be to visibly express sadness in particular contexts. That is, the results of Studies 1 and 2 suggest that while it is seen as okay for men to cry in certain (stereotypically masculine) contexts, they must also be careful to display their tears in what is perceived to be a non-feminine way. For example, Study 1 showed that participants rated crying men as more emotionally appropriate, more emotionally strong, and as having more status in the workplace to the extent that they also perceived those men as masculine. The message that men must “do” tears correctly does not seem to be lost on men themselves, as reflected in the finding from Study 2 that male participants report greater likelihood of crying themselves in weightlifting as compared to figure skating. Overall, then, the results highlight a continued and troubling social hierarchy in which femininity in men is devalued, and in which men who are most closely associated with femininity face social penalties not experienced by men perceived to be more stereotypically masculine.

Given that the results of my studies highlight both, (1) a greater tolerance for men’s stereotypically feminine expressions of emotion than popular stereotypes would suggest, and (2) the continued importance for men of expressing this emotion in a “non-feminine” way, it is clear that more complex and nuanced constructions of men’s emotionality are needed. Such accounts should recognize that men are emotional and expressive beings, while simultaneously acknowledging men’s negotiation of emotional norms within a culture that devalues feminine emotionality.

### Limitations and Directions for Future Research

Although the studies presented here provide an initial test of the idea that men’s crying (and to a lesser extent, women’s) may be viewed most favorably in stereotypically masculine contexts, there remain many important questions to be addressed around this issue. For example, although crying can occur as the result of many different emotions, including sadness, joy, fear, anger, and being “moved” (Vingerhoets, 2013; Zickfeld et al., 2018), the present studies addressed participants’ responses only to sad crying scenarios. Given that most research on tears and crying has focused on perceptions of sad tears, little is known about how tears brought about by other emotions are perceived by observers (see MacArthur and Shields, in press). Therefore, future research should investigate whether similar results would be found if vignette targets were displaying tears of anger, joy, or other tear-eliciting emotions.

Similarly, the present research examined perceptions of crying in scenarios only in relatively serious situations where crying was likely to be seen as warranted (losing an important competition, helping a seriously injured child); therefore, it is also important for future research to investigate whether similar results would be found if the crying scenarios were perceived to be more trivial (e.g,. losing in a non-elimination round of a sports competition).

Another limitation of the present research is that participants’ perceptions of tears in stereotypically masculine settings were compared only to tears in stereotypically feminine settings, and not to a control condition in which the target did not cry. Therefore, the current studies do not tell us whether men in stereotypically masculine contexts are perceived *more* positively when they cry in emotional situations than had they not cried at all, or whether crying in stereotypically feminine settings results in men being downgraded for their tears relative to a situation in which they did not cry (or whether both may be true). Furthermore, although mediation analyses indicated that perceptions of the target’s masculinity were responsible for the results of both studies, these analyses do not rule out the possibility that other factors that vary systematically with masculinity (e.g., degree of physicality) could (at least partially) account for the findings. Therefore, continuing to manipulate masculinity in different ways, as well as determining whether tears have a boosting or buffering effect in stereotypically masculine and stereotypically feminine settings, will be important areas for future research.

Finally, it must be acknowledged that the present research was conducted primarily with White, English-speaking American participants, and using target names that were likely perceived by participants as white. Therefore, it is not known whether results would apply in other racial or cultural contexts. The emotion stereotypes applied to men and women of color are grossly under-researched, but existing work on the “angry black woman” stereotype suggests that tears and crying may be viewed as less typical of women in other racial groups than of white women. For this reason, a critical next step in this research will be to determine existing intersectional stereotypes around tears and crying, as well as how these might inform the results of the present study and crying research in general.

## Conclusion

The present research investigated observers’ perceptions of crying in stereotypically masculine and feminine contexts, and found that men’s (and to a lesser extent, women’s) crying was perceived most favorably in stereotypically masculine contexts, and when coming from individuals perceived to be masculine. Results also showed that men themselves indicated being less likely to cry in a stereotypically feminine versus masculine setting, and that findings that were mediated by perceptions of the vignette target’s masculinity and femininity. Such results align with previous work on masculinity (e.g., Thompson and Pleck, 1986; Connell, 1995), which has suggested that a key way for men to enact masculinity is to avoid behaviors that might be interpreted as feminine. Thus, while the results highlight that stereotypes about men’s relative lack of emotionality are inadequate to capture the reality of men’s tears and crying (and others’ responses to it), they also indicate that cultural expectations for masculinity continue to require men to express emotion in ways that clearly demarcate them from women and femininity. Overall, more nuanced understandings of men’s emotion are needed to account for the complexity of men’s emotional lives.

## Data Availability Statement

The datasets generated for these studies can be found in the Supplementary Material associated with this report.

## Ethics Statement

The studies involving human participants were reviewed and approved by The Pennsylvania State University Institutional Review Board. Written informed consent for participation was not required for this study in accordance with the national legislation and the institutional requirements.

## Author Contributions

HM was responsible for the design and conception of the project, the implementation of data collection, the statistical analyses, and the drafting and revision of the manuscript.

## Conflict of Interest

The authors declare that the research was conducted in the absence of any commercial or financial relationships that could be construed as a potential conflict of interest.

## Supplementary Material

TABLE S1 Full data set for Study 1. For the variable JobIV, 0 = nursing and 1 = firefighting. For the variable GenderIV, 0 = female target and 1 = male target.

TABLE S2 Full data set for Study 2. For the variable Sport, 0 = figure skating and 1 = weightlifting. For the variable TargetGender, 0 = female target and 1 = male target.

## Appendix A: Vignettes

### Nurse Vignette

Jessica works as a nurse in the emergency room of a major city in the U.S. One day when Jessica is on duty, she is told that there’s been a car accident on a road near the hospital, and that a family involved in the accident has arrived at the ER. Jessica meets the ambulance and is the first to attend to a child who is badly injured. Jessica works hard to stabilize the child, who is still breathing and has a heartbeat when she hands him off to the surgical team at the hospital. As she walks back to her nursing station, Jessica feels sad and begins to tear up. Visibly crying, she tells the other nurses, “He’s just a kid.”

### Firefighter Vignette

Jessica works in the fire department of a major city in the U.S. One day when Jessica’s team is on duty, a call comes through the radio that there’s been a car accident on a road near the fire station. Jessica’s team is first on the scene, and Jessica sees that a child in the back seat of one of the cars involved in the accident is badly injured. Jessica works hard to stabilize the child, who is still breathing and has a heartbeat when she hands him off to the paramedics who have arrived on the scene. As she walks back to the fire truck, Jessica feels sad and begins to tear up. Visibly crying, she tells the other firefighters, “He’s just a kid.”

### Sports Vignette

*Jennifer/Jonathan is competing in a national* ***figure skating/weightlifting*** *championship. No one from her/his state has won the competition in over 5 years, and she/he is competing against several of her/his biggest rivals for the title. Throughout the competition, Jennifer/Jonathan performs several successful* ***routines/lifts***, *and feels good about her/his performance as she/he leaves the* ***ice/floor*** *following her final* ***skate/lift***. *Unfortunately, several other* ***skaters/weightlifters*** *score higher than Jennifer/Jonathan in the last round, and when the final results are announced to the crowded arena, she/he does not place in the top three. As she/he reflects on the significance of the championship, Jennifer/Jonathan begins to shed tears. As she/he cries, she/he says, “I really wanted to win.”*
